# Field Testing and Validation of a New Question Set to Measure Housing Status — Fulton County, Georgia, August–September 2023

**DOI:** 10.15585/mmwr.mm7420a2

**Published:** 2025-06-05

**Authors:** Anna Bratcher, Caroline J. Waddell, Christine M. Kava, Hassan Zadeh, Joshua O’Neal, Corinne David-Ferdon, Emily Mosites, Kristie E. N. Clarke

**Affiliations:** ^1^Epidemic Intelligence Service, CDC; ^2^Fulton County Board of Health, Atlanta, Georgia; ^3^National Center for Injury Prevention and Control, CDC; ^4^Office of Readiness and Response, CDC; ^5^Office of Public Health Data, Surveillance, and Technology, CDC.

**Summary**

**What is already known about this topic?**

Housing status data can guide health promotion and effective public health responses. A validated tool to evaluate homelessness and congregate setting residency that aligns with federal definitions and distinguishes sheltered from unsheltered homelessness is not available.

**What is added by this report?**

A new question set to evaluate housing status was field-tested during August–September 2023 among a convenience sample of 481 respondents at food pantries and public health clinics in Fulton County, Georgia. Twenty-six of these respondents were identified in a local housing database; housing status determined by the question set was consistent with data in this database for 24 (92%) respondents, suggesting external validity of the question set.

**What are the implications for public health practice?**

This question set would benefit from validation in additional settings and could help health agencies improve housing data accuracy and consistency, optimizing measures to support persons facing homelessness or living in group settings and their communities.

## Abstract

Although data on housing status can guide health promotion and effective public health response, a validated question set to measure housing status is not available. In June 2023, the Fulton County Board of Health (FCBOH) requested CDC technical assistance to field test a housing status question set for public health case interviews and surveillance. The question set can be asked of any relevant period to determine both homelessness status and residence in a congregate setting. Field testing was performed at food pantries and FCBOH tuberculosis, vaccination, and sexual health clinics in Fulton County, Georgia, during August 2–September 1, 2023. Among 481 respondents who were asked about their living situation during the previous 2 weeks, 139 (28.9%) reported experiencing homelessness and 75 (15.6%) reported living in congregate settings. Twenty-six of these 481 respondents were identified in a local housing database (the Homeless Management Information System [HMIS]); for 24 of these 26 respondents (92%), the housing status recorded in HMIS matched that determined by the question set. The question set would benefit from validation in additional settings and could help health agencies improve housing data accuracy and consistency, optimizing measures to assist persons at higher risk.

## Introduction

Homelessness and congregate setting residence are dimensions of housing status that are particularly important to public health surveillance and action. Persons experiencing homelessness and those living in congregate settings are at higher risk of morbidity and mortality than are those with individual housing (*1*–*3*). Housing status data can guide interventions to promote health and effectively respond to outbreaks (*4*). However, no question set to measure these aspects of housing status has been validated against an independent data source, an important step to ensure that collected data provides a reliable measure of housing status. In June 2023, the Fulton County Board of Health (FCBOH) requested CDC technical assistance to establish a validated housing status question set for public health case interviews and surveillance.

## Methods

Using all combinations of one of the search terms “housing,” “homeless,” “homelessness,” or “unhoused,” and another of the search terms “questions,” “questionnaire,” “screening instrument,” “screener,” or “measurement,” the following internet sites were searched in November 2022 for English-language measurement tools that have been used to determine housing status: U.S. Department of Housing and Urban Development (HUD), Veterans Administration, Centers for Medicare and Medicaid Services, Google Scholar, and PubMed. Partners and subject matter experts in housing and homelessness were also contacted to identify any additional tools. The search retrieved 21 tools, which were then reviewed to determine whether they had undergone validation, were aligned with HUD definitions (*5*) (for comparability with HUD data sources and alignment with federal support programs), and elicited sufficient information for public health use (e.g., ability to differentiate between sheltered and unsheltered homelessness and identify persons housed in a congregate setting).

One of the existing tools had undergone validation with an external data source,* none elicited sufficient detail to align with federal definitions of homelessness (*5**,**6*), and none met public health use case requirements (measurement of sheltered or unsheltered homelessness and congregate or noncongregate living situations). In response, a new question set was developed using an iterative process involving CDC subject matter experts, federal partners, local public health officials, service providers, and clinician–researchers to align with federal definitions and public health use case needs. The question set was also reviewed by CDC subject matter experts and persons with lived experiences of homelessness who serve on the consumer panel of a national partner organization.

### Question Set

The question set (Supplementary Table) included three components that could be asked for any relevant period: 1) whether the respondent stayed in one place or several; 2) open-ended description of the places where the respondent stayed, which the interviewer then matched to a prespecified list; and 3) multiple-choice questions to clarify the housing type, such as whether the respondent’s current arrangement was short- or long-term. Combined answers indicated whether a respondent was housed (i.e., had a fixed, regular, and adequate nighttime residence), was experiencing sheltered homelessness (i.e., staying in emergency shelters, transitional housing programs, or safe havens), or was experiencing unsheltered homelessness (i.e., had a primary nighttime location that is not designated for sleeping accommodations [e.g., streets, passenger vehicles, or parks]). Answers also indicated whether a respondent was living in a congregate setting (i.e., facilities where a majority of persons are not related, living or staying overnight and using shared spaces [e.g., group homes, assisted living facilities, or correctional facilities]) (Box).

BOX **Congregate setting and housing status determination based on housing categories field-tested by Fulton County Board of Health — Fulton County, Georgia, August–September 2023**

**Housed**

**Congregate Setting**

- Residential facility for workers or students
- Correctional or detention facility*
- Facility that provides medical or behavioral health treatment*
- Group home or residential facility not provided by employer or school*
- Multiple^†^

**Noncongregate Setting**

- Private residence in a long-term arrangement
- Hotel or motel or vacation rental in a long-term arrangement
- Multiple^§^

**Sheltered Homelessness**

**Congregate Setting**

- Shelter or safe haven
- Correctional or detention facility^¶^
- Facility that provides medical or behavioral health treatment^¶^
- Group home or residential facility not provided by employer or school^¶^
- Multiple**

**Noncongregate Setting**

- Private residence in a short-term arrangement of ≤14 days^††^
- Hotel or motel or vacation rental in a short-term arrangement of ≤14 days^††^
- Multiple^§§^

**Unsheltered Homelessness**

**Congregate Setting**

- Buildings with shared facilities not meant for human habitation
- Open air, part of an established encampment
- Multiple^¶¶^

**Noncongregate Setting**

- Structure not meant for human habitation
- Vehicle not meant for human habitation
- Open air, not part of an established encampment
- Multiple***

* If stay is ≥90 days regardless of previous situation, or if stay is <90 days and previous situation was not unsheltered homelessness, a shelter, or a safe haven.

^†^ If one or more places is in a congregate setting and is not unsheltered, a shelter, or a safe haven.

^§^ If none of the places is a congregate setting and is not unsheltered, a shelter, or a safe haven.

^¶^ If stay is <90 days and previous situation was unsheltered homelessness, a shelter, or a safe haven.

** If one or more places is in a congregate setting and is a shelter or a safe haven.

^††^ A short-term arrangement is classified as sheltered homelessness only if no subsequent residence is identified (e.g., would not include a person who is currently traveling and has more permanent housing in place). This situation falls under Homelessness Category 2: Imminent Risk of Homelessness as defined by U.S. Housing and Urban Development under the McKinney-Vento Homeless Assistance Act As Amended by S.896 Homeless Emergency Assistance and Rapid Transition to Housing Act of 2009. CoC and ESG Homeless Eligibility - Category 2: Imminent Risk of Homelessness | HUD Exchange

^§§^ If one or more places is in a noncongregate setting and is a shelter or a safe haven.

^¶¶^ If one or more places is in a congregate setting and unsheltered.

*** If one or more places is in a noncongregate setting and unsheltered.

Questions prioritized brevity, with an intended average completion time of <2 minutes. The question set was not geographically specific (i.e., living situations not present or common in Fulton County such as “beach” or “boat” were included in the prespecified list).

### Data Collection

During August 2–September 1, 2023, FCBOH field-tested the question set with CDC technical assistance at food pantries and FCBOH tuberculosis, vaccination, and sexual health clinics in Fulton County. To allow respondents to be matched across data sources to validate housing status data, the question set was embedded within a survey that included demographic information and other identifiers. Teams of two to three interviewers visited clinics each weekday during all operating hours. The number of participants recruited at clinic sites typically varied from 10 to 30; however, one data collection day yielded 78 participants. A team of five to six interviewers visited the food pantry during their 2 operational days per week, recruiting 50 and 35 participants on those 2 days; visits were then discontinued to avoid duplicate responses, because many clients returned weekly to the pantry. Respondents received a $10 gift card to their choice of a pharmacy or grocery store. The survey collected data on personal identifying information, demographic characteristics, and responses to the housing question set. Interviewers conducted the survey and then noted the respondent’s engagement level and comfort with answering the questions (i.e., acceptability) and recorded any challenges with administering the survey. Both electronic and paper versions of the questions were tested. Interpretation services were available to all respondents, either by testing site personnel, an accompanying family member, or a CDC interpretation phone line with interpretation capabilities for 171 languages. Interview feasibility was determined by the ability to provide the question set to a wide range of clients without substantial difficulty. Interviewers recorded subjective impressions of respondent engagement with five options: 1) very hesitant or distracted, 2) somewhat hesitant or distracted, 3) neutral, 4) somewhat engaged and willing, and 5) fully engaged and willing to answer questions. This activity was reviewed by CDC, deemed not research, and was conducted consistent with applicable federal law and CDC policy.^†^

### Validity Analysis

The internal validity assessment compared data gathered from respondents, recruited at the same time, who reported staying at the same. Surveys for these respondents were completed separately, and answers were later linked and compared. The local Homelessness Management Information System (HMIS) was used as an independent comparator to measure external validity (*7*). Despite low coverage of all persons experiencing homelessness (*8*), HMIS is the most comprehensive client-level data source for persons in the covered area who receive housing-associated program (e.g., rent vouchers, street outreach, and homeless shelters) or auxiliary (e.g., food support, laundry, or shower) services funded by HUD.

Survey respondents were included in the external validation analysis if 1) a service that the respondent received was recorded in HMIS as related to housing type (e.g., housing vouchers, homeless shelter stays, or outreach at an encampment), and 2) data in HMIS were within 2 weeks before the survey date or the most recent HMIS entry before the survey date was listed as stable housing, under the assumption that this housing status was accurate at the time of the survey. Survey and HMIS records were matched on full name and birth date, accounting for common typographical errors in manual entry fields (i.e., fuzzy matching^§^) to determine agreement in reported housing status between the two. Data analysis was conducted using SAS software (version 9.4; SAS Institute).

## Results

### Acceptability and Feasibility

A convenience sample of 481 respondents completed the survey (Table). Twenty-six surveys (5.4%) were conducted in a language other than English: Spanish (4.6%), Creole (0.4%), Korean (0.2%), and Mandarin (0.2%). Among 478 participants (three had missing observations), interviewers recorded that 99.2% were somewhat or completely engaged when answering the questions. Although some participants declined to answer certain personal information and demographic questions, no respondent declined to answer or expressed reservation about answering questions in the housing question set. One survey was not completed due to time; the participant was called for their clinical visit during survey completion.

**TABLE:** Characteristics of respondents in field testing of question set to measure housing status — Fulton County, Georgia, August–September 2023

Characteristic	No. (%)
**Total**	**481 (100)**
**Age group, yrs**	
18–29	70 (14.6)
30–39	138 (28.7)
40–49	96 (20.0)
50–59	82 (17.0)
60–69	71 (14.8)
≥70	19 (3.9)
Declined to answer	5 (1.0)
Race and ethnicity*	
American Indian or Alaska Native	18 (3.7)
Asian	8 (1.7)
Black or African American	382 (79.4)
Hawaiian or Pacific Islander	0
Hispanic, Latino, or of Spanish origin	55 (11.4)
White	52 (10.8)
No race provided^† ^	6 (1.2)
Other	40 (8.3)
**Sex^§^**	
Female	201 (41.8)
Male	276 (57.4)
Other	4 (0.8)
**Language in which survey was completed**	
English	455 (94.6)
Creole, via family member interpreter	2 (0.4)
Korean, via phone line	1 (0.2)
Mandarin, via phone line	1 (0.2)
Spanish, via staff member interpreter	18 (3.8)
Spanish, via phone line	4 (0.8)
**Survey site**	
Tuberculosis, vaccination, and sexual health clinics	396 (82.3)
Food pantry	85 (17.7)
**Engagement and willingness of the respondent^¶^**	
Fully engaged and willing to answer questions	456 (95.4)
Somewhat engaged and willing	18 (3.8)
Neutral	1 (0.2)
Somewhat hesitant or distracted	2 (0.4)
Very hesitant or distracted	1 (0.2)
Missing**	3 (—)
**Housing status**	
Housed	331 (68.8)
Sheltered homelessness	56 (11.6)
Unsheltered homelessness	58 (12.1)
Both sheltered and unsheltered homelessness	25 (5.2)
Unclear	11 (2.3)
**Living in a congregate setting?**	
Yes	75 (15.6)
No	406 (84.4)

* Not mutually exclusive.

**^†^** Explicitly selected by the respondent.

^§^ Sex was self-reported at the time of the survey; four participants self-reported an identity other than male or female sex.

^¶^ Recorded by interviewer.

** Missing responses were not included in the denominator.

Electronic (n = 20) and paper (n = 461) versions performed equally well in respondent engagement. Interviewers reported being initially uncertain when selecting from the prespecified list of housing types; once familiar with this list, their confidence increased.

During the 2 weeks before the survey, 331 (68.8%) respondents reported being housed and 139 (28.9%) experienced homelessness; housing determination was unclear for 11 (2.3%) respondents who were uncertain about the expected length of time of their current living arrangements. Among the 139 respondents experiencing homelessness, 56 (40.3%) were sheltered, 58 (41.7%) were unsheltered, and 25 (18.0%) had stayed in both sheltered and unsheltered locations. Among all respondents, 75 (15.6%) reported living in congregate settings (e.g., residential facility, shelter, or safe haven).

### Validity Analysis

In the assessment of internal validity, among respondents staying together, 20 of 21 paired survey responses (95.2%) resulted in the same classification for housing and congregate residence status. Twenty-six respondents (5.4%) met inclusion criteria for the external validity analysis; these respondents collectively gave responses corresponding to 10 of the 14 housing types (Box). The housing status determined by the question set was consistent with the HMIS status for 24 of these respondents (92.2% concordance; 18 experiencing homelessness, and six housed). When responding to the questions, two respondents were recorded as housed in the most recent HMIS entry but reported at least one living situation meeting the definition of homelessness during the previous 2 weeks.

## Discussion

The housing status question set demonstrated acceptability, feasibility, and validity when field tested in Fulton County, Georgia. Among a small subset of respondents identified in a local HMIS, agreement between the tested question set and HMIS data was approximately 90%. However, future activities might try to confirm this external validity and expand assessment of the question set to other geographic populations. To increase feasibility, it is important that interviewers become familiar with the prespecified options for housing types and the names of local shelters and safe havens before use in the field. Data standards are necessary for meaningful exchange of health-related information between modern data systems; therefore, a corresponding data standard is needed for housing status. CDC is working with partners to develop such a standard for consideration by organizations overseeing standard data classes and elements used in systems across the United States, such as the U.S. Core Data for Interoperability.

### Limitations

The findings in this report are subject to at least five limitations. First, question set acceptability, feasibility, and validity might not generalize to other settings outside of Fulton County, Georgia. Second, the external validation analysis relied on the matching of survey data to HMIS, which was limited to approximately 5% of respondents. Errors in name and birth date in both data sets might have contributed to the low proportion of responses eligible for matching. Third, external validation analysis included responses corresponding to 10 of the 14 housing types; the same level of validity might not apply to the remaining types. Fourth, internal validation was restricted to respondents who cohabitated or lived in a congregate setting and might not be generalizable to those who live alone. Finally, although feasible and acceptable, integration of this question set into existing public health, social services, and clinical workflows and data systems might be a challenge because training is required to administer the question set effectively. This concern could be addressed through more user-friendly approaches such as those including a free-text interface with automated selection from the prespecified housing list (e.g., application of a large language model).

### Implications for Public Health Practice

This question set provides the first approach to determining housing status shown to be externally valid for a small subpopulation of respondents; all respondents reported as unhoused in HMIS were determined to be unhoused by the question set. Thus, this tool might help health agencies and other organizations screen for homelessness. Standardized and accurate housing data allow public health practitioners to quickly and efficiently focus activities to assist groups at higher risk for adverse outcomes, particularly during a public health emergency. This tool might also be valuable in health care settings because housing status affects both individual and population health. Because data are increasingly integrated across systems, consistency in how information is collected and transmitted is an important component to optimization of data quality.

## Footnotes

*
Informatics for Health: Connected Citizen-Led Wellness and Population Health | IOS Press

† 45 C.F.R. part 46.102(l)(2), 21 C.F.R. part 56; 42 U.S.C. Sect. 241(d); 5 U.S.C. Sect. 552a; 44 U.S.C. Sect. 3501 et seq.

§ Fuzzy matching is a data matching technique used to find approximate matches for strings, phrases, or words in a database when an exact match is not available.
